# Expected Values of Artificial Intelligence in Colon Capsule Endoscopy: Artificial Intelligence in Capsule Endoscopy Consortium Position Statement

**DOI:** 10.1055/a-2905-8231

**Published:** 2026-08-24

**Authors:** Konstantinos Triantafyllou, Georgios Tziatzios, Alexandra Agache, Ramesh Arasaradnam, Begoña González Suárez, Martin Keuchel, Kirsten O. Kyvik, Benedicte Schelde-Olesen, Xavier Dray, Reena Sidhu, Gunnar Baatrup, Anastasios Koulaouzidis, AICE Consortium Work Package 5 Task Force Working Group Task Force Working Group, Victoria Blanes-Vidal, Jan Matthias Braun, Cristina Carretero, Pablo Cortegoso Valdivia, Ulrik Deding, Madeleine Hayenhjelm, Ian Io Lei, Wojciech Marlicz, Miguel Mascarenhas, Maroulla Nikolaki, Lea Østergaard Hansen, Bruno Rosa, Ervin Toth, James Turvill, Angus Watson, Thomas Bjørsum-Meyer

**Affiliations:** 1 Department of Gastroenterology, Medical School, National and Kapodistrian University of Athens 69038 General University Hospital AttikonAthensGreece; 2 Department of GastroenterologyGeneral Hospital of Nea Ionia “Konstantopoulio-Patision”AthensGreece; 3 Department of Surgery 11286 Odense University Hospital and Svendborg SygehusSvendborgSyddanmarkDenmark; 4 Department of Clinical Research 6174 University of Southern DenmarkOdenseDenmark; 5 Department of General Surgery 87267 University of Medicine and Pharmacy Carol Davila BucharestBucharestRomania; 6 GastroenterologyUniversity Hospitals Coventry and Warwickshire NHS TrustCoventryUnited Kingdom of Great Britain and Northern Ireland; 7 Gastroenterology Department, ICMDM, Hospital Clínic. University of Barcelona, Spain; August Pi i Sunyer Biomedical Research Institute (IDIBAPS), Barcelona, Spain; CIBEREHD, Centro de Investigación Biomédica en Red de Enfermedades Hepáticas y Digestivas, Instituto de Salud Carlos III, MadridSpain; 8 Clinic for Internal Medicine, Agaplesion Bethesda Hospital Bergedorf, HamburgGermany; 9 Research Support Center 72750 Region ZealandSorøRegion ZealandDenmark; 10 Sorbonne University, Centre for Digestive Endoscopy, Saint Antoine Hospital, APHP, Paris; Liver & Microbiome Research (GLIMMER) FHU, ParisFrance; 11 Division of Clinical Medicine, School of Medicine and Population HealthUniversity of SheffieldSheffieldEnglandUK; 12 Sheffield Teaching Hospitals NHS Trust & University of SheffieldSheffieldUK; 13 The Maersk Mc-Kinney Moller Institute, University of Southern Denmark, OdenseDenmark; 14 Department of Gastroenterology, Clínica Universidad de Navarra, Pamplona, NavarraSpain; 15 Gastroenterology and Endoscopy Unit, University Hospital of ParmaParmaItaly; 16 Faculty of Arts, Department of Historical, Philosophical and Religious Studies, Umeå UniversityUmeåSweden; 17 Institute of Precision Diagnostics and Translational Medicine, University Hospitals Coventry and Warwickshire NHS TrustCoventryUK; 18 Warwick Medical School, University of WarwickCoventryUK; 19 Department of Gastroenterology, Pomeranian Medical UniversitySzczecinPoland; 20 Hospital São João Porto and Faculty of Medicine of the University of PortoPortoPortugal; 21 Gastroenterology Department, Hospital da Senhora da Oliveira–Guimarães, Unidade Local de Saúde do Alto Ave (ULS Alto Ave), GuimarãesPortugal; 22 Life and Health Sciences Research Institute (ICVS), School of Medicine, University of Minho, BragaPortugal; 23 Department of Gastroenterology, Skåne University Hospital, Lund UniversityMalmöSweden; 24 York and Scarborough Teaching Hospitals NHS Foundation TrustYorkUK; 25 Department of General Surgery, NHS HighlandInvernessUK; 26 Department of General Surgery, University of AberdeenAberdeenUK

**Keywords:** artificial intelligence, colon capsule endoscopy, colorectal cancer, colorectal polyps, screening, quality indicators

## Abstract

The purpose of this position statement is to propose expected performance values for artificial intelligence (AI) applications in colon capsule endoscopy (CCE), with emphasis on detection, characterization, localization, reporting, and downstream management of colonic neoplasia. The task force was created within the Artificial Intelligence in Capsule Endoscopy (AICE) consortium project work and drew on members of the international CApsule endoscopy REsearch (iCARE) group for development, review, consensus voting, and final approval. These values are intended as consensus-based benchmarks for research, validation, and future clinical translation, while explicitly acknowledging the limited availability of robust CCE-specific clinical evidence. The task force included CCE experts, technical experts, patient representatives, and an ethicist, and defined expected values using existing endoscopy quality frameworks, available capsule endoscopy literature, relevant proxy evidence from colonoscopy, and structured Delphi consensus. A supermajority agreement of more than 80% across up to three Delphi voting rounds was required for consensus. The agreed statements address candidate selection, assessment of mucosal visualization and procedure completeness, landmark and segment recognition, polyp matching, lesion size estimation, reading-time reduction, detection of colorectal neoplasia, false-positive burden, risk stratification for subsequent procedures, prediction of advanced pathology, urgency categorization, and automated report generation. The proposed thresholds should not be interpreted as definitive requirements for immediate clinical adoption. Instead, they identify minimum expected value and aspirational value that future systems should be tested against in prospective, externally validated, full-video CCE studies.

## Source and Scope

Created within the Artificial Intelligence in Capsule Endoscopy (AICE) consortium project work and drawing on members of the international CApsule endoscopy REsearch (iCARE) group, this position statement outlines expected values for artificial intelligence (AI) tasks in colon capsule endoscopy. It should be read as a forward-looking consensus and research-prioritization framework rather than as a formal evidence-based clinical guideline. The focus is on the diagnosis and management of colonic neoplasia and on the potential impact of AI on existing or emerging quality performance measures defined by the European Society of Gastrointestinal Endoscopy and other international societies.

## Introduction and Objectives

Compared to breast, cervical, and prostate cancer screening programs, participation in colorectal cancer (CRC) screening programs remains relatively low. 1 Noninvasive alternatives to conventional colonoscopy, such as colon capsule endoscopy (CCE), are being explored to enhance screening efficiency, minimize unnecessary invasive procedures, and improve patient experience. The use of CCE examination can aid in prioritizing individuals with polyps, thereby managing demand more effectively. One strategy involves prioritizing colonoscopic intervention for individuals with large polyps measuring ≥10 mm. Conversely, individuals with diminutive (<6 mm) or small (6–9 mm) polyps can safely follow routine pathways, resulting in a downgrade in priority. 2

A comprehensive summary of seven systematic reviews on CCE implementation in CRC screening, published from 2016 to 2022, revealed that for the detection of polyps measuring ≥10 mm, the pooled sensitivity (Se) and specificity (Sp) of second-generation CCE (CCE2) ranged from 84% to 94% and from 85% to 96%, respectively. Examination completion and adequate cleanliness rates ranged from 57% to 100% and 61% to 92%, respectively. Additionally, in six studies comparing CCE performance to CT colonography, the Se of CCE was consistently higher for detecting small, flat lesions. 3 Although these results may indicate CCE as an attractive alternative to colonoscopy, challenges exist in transitioning to CCE-oriented CRC screening, including resistance to change, concerns about accuracy and reliability, financial considerations, implementation costs, training requirements, and the absence of standardized guidelines, regulations, and reimbursement structures. These factors collectively hinder the widespread adoption of CCE. 3

Artificial intelligence (AI) represents a significant advancement in diagnostic endoscopy, aiding endoscopists in specific, well-defined tasks such as detecting and characterizing gastrointestinal (GI) neoplasia. 4 5 The European Society of Gastrointestinal Endoscopy (ESGE) issued a Position Statement in 2022 emphasizing the anticipated value of AI for GI procedures, although CCE was not included in the list of endoscopic modalities. The authors underscore that the benefits and risks of AI can be forecast based on the clinical relevance of the intended task and preliminary evidence from artificial or clinical environments. 6 According to this Position Statement, the “expected value” of AI (essentially, the value anticipated before well-designed clinical trials) is contingent upon the clinical implications of endoscopic performance limitations and AI's potential to address these. Factors influencing this expected value include the potential harms of AI, such as the ramifications of false-positive results or AI-induced deskilling of endoscopists. 7 Furthermore, the endoscopist’s training and expertise, the prevalence and severity of diseases, the interaction between AI and human endoscopists, and the costs of implementation are all crucial factors. In addition, establishing clear reference standards is imperative for delineating AI’s value in managing GI neoplasia. 6

Assessing the expected value of AI-related tasks is most effectively done within the framework of existing quality performance measures delineated by ESGE for colonoscopy and CCE procedures. 8 9 This approach offers clear definitions, measurement methods for each indicator, and clinically relevant cutoff points where applicable. Such a framework also facilitates transparent evaluation of AI systems’ benefits and risks in clinical practice, preventing redundant performance measure assessments. Nevertheless, due to the novelty of AI, it is plausible that some AI-related tasks may not be covered by existing performance measures, necessitating the establishment of new AI-centric performance measures.

The objective of this position statement is to outline AI tasks within established and emerging performance-measure domains, and to propose minimum expected value and aspirational value for future validation in CCE practice. These values are consensus-based benchmarks and research priorities, not definitive clinical implementation standards. The emphasis is on clinical rather than purely technical validation, with a primary focus on the detection, characterization, localization, reporting, and downstream management of colonic neoplasia.

## Methodology

The task force was created within the Artificial Intelligence in Capsule Endoscopy (AICE) consortium project work and drew on members of the international CApsule endoscopy REsearch (iCARE) group for development, review, consensus voting, and final approval (Supplemental file 1). It included clinical, technical, ethical, and information experts, as well as patient representatives, to assess the expected value of AI systems in CCE. The task force identified specific domains and delineated the AI tasks within each domain ( Table 1 ). To align AI tasks with performance measures and define their expected values, the existing methodology 6 was adopted, including:

- **Task definition** : Main specific and operational tasks performed by AI systems (e.g., assessing bowel preparation level, recognizing and localizing colorectal lesions) were identified through targeted narrative literature searches, incorporating both artificial and clinical studies. Without high-quality data, values were either extrapolated from existing literature (e.g., conventional colonoscopy) or developed through consensus.
- **Integration of AI tasks with performance measures** : Each AI task was categorized into one of the eight corresponding domains, such as preprocedure, completeness of procedure, identification of pathology, management of pathology, complications, patient experience, postprocedure, and ethics. For each task, outcomes in terms of benefit and harm were described. When relevant, established ESGE performance measures were considered. New performance measures were developed when AI tasks did not align with existing quality standards.
- **Reference standard** : Clinically relevant reference standards were defined for each outcome against which to assess the potential impact of AI tasks. The term “comparable with the reference standard” was generally used to express equivalent performance, with “experienced” and “less experienced” endoscopists referenced throughout the text.
- **Expected value assessment:** An expected value for the AI performance measure was defined for each outcome based on the clinical relevance of the performance measure related to the AI task and the expected benefits and harms of AI implementation.
- Literature search and threshold derivation: Due to the paucity of evidence in AI-assisted capsule endoscopy (CE), a targeted narrative literature search was undertaken. Thresholds were informed by available CCE and CE literature where directly relevant, cautious extrapolation from established colonoscopy and endoscopy performance-measure frameworks where appropriate, clinical judgment regarding the potential benefits and harms of AI implementation, and structured Delphi consensus. Consequently, the proposed values should be interpreted as expected-value benchmarks for research and validation rather than as definitive evidence-based clinical requirements.
- **Delphi agreement** : Up to three rounds of voting were anticipated. A participation rate of more than 75% was a prerequisite for a valid voting round. A supermajority agreement of >80% was required for consensus on each statement. If consensus was not reached, the task force members reviewed and adjusted the measures, leading to additional rounds of voting.
- **Maintenance of this Position Statement** : The development of this statement involves an ongoing targeted narrative review of the literature and relevant trials. Importantly, this document will be a dynamic, living document. This means that the task force will continuously review new literature and trial results. Depending on the accumulation of data, they will make necessary amendments and, if applicable, formulate PICO questions. Subsequently, they will conduct meta-analyses to develop guideline recommendations, utilizing the Grading of Recommendations Assessment, Development and Evaluation methodology to ensure scientific rigor, visibility, and global reach. 10 Additionally, they will take on the responsibility of monitoring new data accumulation and will update the living document regularly to reflect the latest advancements and insights in the field of AI in CCE.

**Table 1:** Domains and AI tasks for CCE.

Domain	1. Preprocedure	2. Completeness of procedure	3. Identification of pathology	4. Management of pathology	5. Complications	6. Patient experience	7. Postprocedure	8. Ethics
AI Tasks	Pre-CCE assessment: prediction of successful CCE examination, including complete colorectal transit and adequate bowel preparation AI-based candidate selection for CCE.	Assessment of complete mucosal visualization Identification of colon landmarks and colon segments Correct assignment of pathology to colonic segments Polyp localization and CCE path reconstruction Visualization of the small bowel.	Reading time. Polyp matching to avoid double reporting. Polyp size measurement. Se and Sp for: CRC. Polyps ≥6 mm. Polyps ≥10 mm. Polyps, overall. False-positive rate Nonneoplastic pathology detection rate.	AI-assisted risk stratification for downstream management after CCE (PIVI/SODA as indirect safety benchmarks, not directly transferable CCE standards) CCE prediction of submucosal invasion (long-term research objective).	CCE retention. Colonic perforation.	1. AI recognition or recording of patients’ experience before, during, and after CCE. 2. Differences in the recordings by AI (e.g.) experience with different preparation, setting, and outcome, etc.	1. AI-generated (populated) report. 2. AI-generated alerts and interfaces with clinicians and patients	1. AI-related ethics issues. Trust, explainability, etc.

AI: Artificial intelligence; CCE: Colon Capsule Endoscopy; Se: Sensitivity; Sp: Specificity; PIVI: Preservation and Incorporation of Valuable Endoscopic Innovations; SODA: Simple Optical Diagnosis Accuracy.

## DOMAIN. Preprocedure

### AI Task 1.1—Pre-CCE Assessment: Prediction of Successful CCE Examination

The proposed framework for AI-assisted prediction of successful CCE examination is summarized in Table 1.1 .

**Table 1.1:** Prediction of CCE Success.

Technique	CCE.
Domain	Preprocedure.
AI task	To predict a successful CCE examination, meaning complete colorectal transit with adequate bowel preparation in all colonic segments.
Description	AI-assisted prediction of successful CCE examination.
Performance measure	Rate of predicted successful CCE examination.
Rationale	To develop a high-performing model to accurately predict which patients are likely to have a combination of an acceptable bowel preparation and complete colorectal visualization. This reduces the need for reinvestigation due to incomplete tests or poor views.
Reference standard	Meta-analyses of patient predictors of test completion and bowel preparation for CCE.
Expected value	AI-assisted prediction of successful CCE should be ≥85% with an aspirational value of ≥90%.

**STATEMENT**

**For acceptance of AI in clinical practice, the model should predict CCE success in terms of examination completion and adequacy of preparation in at least 85% of the cases.**

Agreement: 100.0%, at the first voting round.

## Discussion

CCE provides a noninvasive alternative to colonoscopy but is constrained by its dependence on passive transit and bowel preparation. 11 Without suction, lavage, or intervention, failure to complete the exam or poor visualization renders the test nondiagnostic. 12 13 14 15 16 Consequently, patient selection is critical. Demographic and clinical characteristics may predict CCE outcomes, and AI can exploit these factors to build accurate prognostic models. 17 18 19 20

Data from the NHS Highland cohort (n=401) identified statistically significant but weak associations between demographics and CCE outcome. 17 A gradient boosting classifier outperformed other models but was insufficiently accurate for clinical deployment. Expanded datasets from 3000–4000 patients across NHS Scotland are under evaluation and may improve AI robustness.

Such tools could triage patients toward CCE or conventional colonoscopy, based on predicted examination success. This strategy could streamline diagnostics and reduce the burden of failed CCEs. The balance of missed diagnoses versus resource optimization justifies 85–90% prediction accuracy thresholds.

## DOMAIN. Preprocedure

### AI Task 1.2—AI-based Candidate Selection for CCE

The proposed AI-driven framework for safe triage to CCE is summarized in Table 1.2 .

**Table 1.2:** AI-assisted candidate selection for CCE.

Technique	CCE.
Domain	Preprocedure.
AI task	Identification of patients who are optimal candidates for CCE.
Description	AI-based preprocedure model to exclude patients at high risk of significant colonic pathology (e.g., advanced neoplasia [by size or histology], CRC or multiple adenomas), enabling safe triage to CCE instead of conventional colonoscopy.
Performance measure	Negative predictive value (NPV) for the absence of clinically significant pathology.
Rationale	The principal utility of CCE is in patients unlikely to harbor high-risk findings. AI can support selection by identifying low-risk individuals using clinical, demographic, and biomarker data, minimizing missed pathology, and optimizing CCE deployment.
Reference standard	Colonoscopy or validated CCE outcomes annotated for pathological significance (e.g., adenoma size, number, dysplasia).
Expected value	NPV ≥ 95% for absence of significant pathology. Sensitivity and specificity metrics should support safe clinical use. Thresholds tuned to local population risk profiles.

**STATEMENT**

**AI models for CCE triage should achieve a NPV of at least 95% for ruling out significant colonic pathology to be acceptable for clinical implementation.**

Agreement: 87.5%, at the first voting round.

## Discussion

CCE is best suited to patients with a low probability of significant colorectal findings, 2 16 21 , as detailed in Table 1.2.1 . Its inability to biopsy or perform therapy, and its limitations in identifying subtle lesions under suboptimal conditions, make it an inappropriate test in high-risk populations. 3 Therefore, a key challenge is identifying low-risk individuals who can safely undergo CCE without compromising diagnostic safety. 3 Recent models by Lei et al. and others have demonstrated that combining structured data (e.g., age, sex, anemia, fecal immunochemical test [FIT] level, symptoms) can reliably exclude advanced neoplasia. Lei’s algorithm achieved an NPV >95% for the absence of significant pathology, supporting its use as a filter before CCE referral. 22 Evidence from the ScotCap program reinforces this principle. 17 In large-scale evaluations, patients with low FIT (<20 μg/g), no anemia, and normal inflammatory markers had a very low yield of clinically significant lesions, suggesting they are ideal candidates for noninvasive testing. 12 15 When these criteria were applied in pilot triage models, unnecessary colonoscopies were substantially reduced without compromising safety.

**Table 1.2.1:** Detailed description of significant and nonsignificant findings in CCE.

Category	Finding	Classification	Need for follow-up/Intervention	Timing/Urgency	Rationale/Notes
Neoplasia	CRC	Significant	Yes	Immediate	Requires immediate intervention (colonoscopy ± surgery).
Neoplasia	Adenoma ≥10 mm	Significant	Yes	Nonurgent (3-year surveillance if complete removal)	Advanced adenoma per ESGE/BSG; surveillance colonoscopy at 3 years if fully excised.
Neoplasia	Villous or tubulovillous histology (≥25%)	Significant	Yes	Nonurgent (3-year surveillance)	Advanced histology (villous component); ESGE recommends 3-year surveillance if ≥10 mm or advanced features.
Neoplasia	High-grade dysplasia	Significant	Yes	Nonurgent (3-year surveillance)	Premalignant; ESGE/BSG surveillance at 3 years after complete removal.
Neoplasia	Multiple adenomas meeting guideline-defined surveillance criteria	Significant	Yes	Nonurgent (3-year surveillance)	Meets high-risk surveillance criteria (ESGE: ≥5 adenomas = 3 years; BSG: ≥2 premalignant with advanced features). Note: Threshold varies slightly by guideline.
Serrated Pathway Lesions	Sessile serrated lesion ≥10 mm or with dysplasia	Significant	Yes	Nonurgent (3-year surveillance)	Elevated CRC risk; ESGE/BSG/USMSTF recommend 3-year surveillance for advanced serrated lesions.
Serrated Pathway Lesions	Traditional serrated adenoma	Significant	Yes	Nonurgent (at 3-year surveillance)	Considered advanced serrated/pre-malignant; ESGE recommends 3-year follow-up.
Serrated Pathway Lesions	Small hyperplastic polyp (left colon)	Nonsignificant	No	None (return to screening)	Common, benign; no increased CRC risk per ESGE/WASP classifications.
Inflammatory Changes	Features suggestive of active IBD	Significant	Yes	Urgent (histology + treatment)	Needs histological confirmation; potential active IBD requiring therapy.
Inflammatory Changes	Mucosal ulceration/friability	Significant	Yes	Urgent	Possible IBD, ischemia, or neoplasia; warrants colonoscopy/biopsy.
Inflammatory Changes	Mild, focal erythema without other features	Nonsignificant	No	None	Often nonspecific; not actionable without additional signs.
Vascular Lesions	Angiodysplasia with bleeding or anemia	Significant	Yes	Nonurgent to Urgent (if symptomatic)	May require endoscopic treatment (e.g., argon plasma coagulation) if symptomatic.
Vascular Lesions	Incidental angiodysplasia, nonbleeding	Nonsignificant	No	Observation	Typically, no intervention; monitor if asymptomatic.
Others	Normal CCE with no mucosal abnormalities	Nonsignificant	No	None (reassuring)	Reassuring result; return to screening pathway.

CRC: colorectal cancer; ESGE: European Society of Gastrointestinal Endoscopy; BSG: British Society of Gastroenterology; USMSTF: U.S. Multi-Society Task Force on Colorectal Cancer; IBD: Inflammatory bowel disease; WASP: Workgroup Serrated Polyps and Polyposis (WASP); CCE: colon capsule endoscopy.

Histology-based categories and surveillance intervals apply only after confirmatory colonoscopy and complete resection. A significant lesion detected by CCE should first be referred for colonoscopy.

Preliminary results from institutional work show that integrating clinical history and patient characteristics into multivariate models yields high predictive performance for CCE completion and advanced neoplasia detection. 23 24 These models could safely redirect up to 40% of lower-risk referrals to CCE. 25 Furthermore, de Jonge et al. 26 have proposed a risk stratification model for FIT-positive patients, identifying a subset with low-risk profiles who could avoid immediate colonoscopy. This concept aligns well with AI-enhanced triage to CCE as a first-line modality in selected patients. 5

Such models must be prospectively validated and tailored to local referral populations. Interpretability is essential; clinicians must understand and justify why a patient is deemed suitable for CCE, particularly if colonoscopy is deferred. Where AI can provide transparent, reproducible predictions, it has the potential to become a powerful tool for safe and efficient diagnostic stratification in colorectal services. 6 27

## DOMAIN. Completeness of procedure

### AI Task 2.1—Proportion of Complete Mucosal Visualization

The proposed framework for AI-assisted assessment of colonic mucosal visualization is summarized in Table 2.1 **.**

**Table 2.1:** Mucosal visualization in CCE.

Technique	CCE.
Domain	Completeness of procedure.
AI task	Assessment of complete mucosal visualization.
Description	Real-time AI-assisted CCE reader assessment of completeness of mucosal visualization.
Performance measure	Assessment of the proportion of complete mucosal visualization and segment-specific adequacy of views.
Rationale	The diagnostic accuracy of CCE is closely related to complete mucosal visualization. Incomplete visualization increases the risk of missed lesions, necessitating further investigation or repeat testing. AI can calculate mucosal coverage metrics and potentially deliver real-time image quality assessments, optimizing reading time and standardizing performance across operators and sites. By automating the identification of nonvisualized segments, AI enhances decision-making by identifying cases that may need additional procedures and reducing the risk of providing false reassurance. Beyond the full length of the CCE video, AI output must include segment-specific mucosal visualization percentages and identify nonvisualized regions using validated thresholds.
Reference Standard	Visual estimation of mucosal visualization by experienced CCE readers, using semiquantitative and segmental scoring systems.
Expected value	Minimum expected value: AI-assisted assessment of colorectal mucosal visualization should achieve ≥90% concordance with expert-reader consensus. Aspirational value: ≥95% concordance with expert-reader consensus.

**STATEMENT**

**For the acceptance of AI in evaluating the completeness of mucosal visualization, AI-assisted assessment should be at least comparable with expert-reader consensus for the full-length CCE video.**

Agreement: 83.3%, at the first voting round.

## Discussion

A clear view of the colonic mucosa is essential for diagnostic confidence in CCE. Unlike colonoscopy, CCE does not permit real-time adjustments such as suction, lavage, or repositioning. Therefore, proper bowel preparation and subsequent mucosal visualization are crucial for successful procedures and avoiding unnecessary downstream interventions. For colonoscopy, ESGE recommends that adequate bowel preparation and unadjusted cecal intubation should each be achieved in at least 90% of procedures, with target standards of at least 95%; however, these procedure-level performance measures do not represent a quantified threshold for the proportion of colonic mucosa visualized. 8

AI-based systems are increasingly capable of segmenting colon capsule videos to estimate mucosal visualization across colonic regions. 28 This technology leverages machine learning (ML) models trained to distinguish visual features suggestive of clear or obscured mucosa. Several pilot studies have shown that AI models can match or surpass human-level assessment. 29 30 31 32

Currently, reference standards rely on assessments by experienced readers using validated semiquantitative scoring systems, such as the Leighton-Rex 33 or CC-CLEAR scales. 34 While these methods are reliable, they remain subjective and prone to interobserver variability, with an overall interobserver agreement of 0.84 (0.82–0.85) for the Leighton-Rex scale and 0.73 (0.71–0.76) for the CC-CLEAR scale. 35 AI may not only complement human reading but also standardize and potentially replace subjective visual estimates by offering consistent, reproducible, and explainable metrics.

Prospective validation across multi-institutional datasets is necessary before broad implementation. Ideally, AI outputs should be interpretable by clinicians, displaying per-segment visualization maps and clarity confidence scores. In future iterations, visualization assessment could be integrated with lesion-detection probability scores to generate a comprehensive adequacy index for clinical use.

## DOMAIN. Completeness of procedure

### AI Task 2.2—Identification of Colon Landmarks

The expected role of AI in colonic landmark identification is summarized in Table 2.2 **.**

**Table 2.2:** Identification of colonic landmarks in CCE.

Technique	CCE
Domain	Completeness of procedure
AI task	Identification of colonic landmarks
Description	Real-time AI-assisted CCE reader identification of colonic landmarks
Performance measure	Correct identification of colonic landmarks
Rationale	Identifying colonic landmarks is crucial for evaluating procedural completeness, assigning segmental cleansing scores, and locating pathology. Landmark recognition remains operator-dependent, with poor interobserver agreement, limiting reliability. AI can assist in standardizing the identification and documentation of key anatomical sites, including the ileocecal valve, appendiceal orifice, cecum, hepatic flexure, splenic flexure, rectum, and anal verge. This can guide downstream procedures, reduce reader fatigue, improve lesion localization, and ultimately reduce repeat testing and procedure time. AI systems must provide interpretable outputs documenting landmark recognition with timestamps, segment labels, and confidence metrics
Reference Standard	Expert-reader consensus for identification of colonic landmarks by experienced CCE readers
Expected value	Minimum expected value: AI-assisted identification of colonic landmarks should reach ≥90% concordance with expert-reader consensus. aspirational value: ≥95% AI concordance with expert-reader consensus.

**STATEMENT**

**For the acceptance of AI in evaluating colonic landmark identification, AI-assisted assessment should be at least comparable to that of experienced CCE readers.**

Agreement: 86.4%, at the second voting round.

## Discussion

The effectiveness of CCE relies not only on proper bowel preparation but also on precise anatomical localization. Identifying key landmarks—such as the ileocecal valve, appendiceal orifice, hepatic flexure, splenic flexure, rectum, and anal verge—is essential for ensuring complete coverage of the colon, assessing segmental cleansing, and pinpointing any pathology. 36 37 38 Given the passive nature of CCE and its lack of navigational control, readers rely on visual cues to estimate anatomical progression.

However, interobserver agreement for landmark identification is low. Schelde-Olesen et al. (2023) reported an agreement rate of only 51% among trained readers, demonstrating the critical need for standardization. 35 AI offers a promising solution by applying feature recognition and spatiotemporal analysis to automate landmark detection.

Herp et al. (2021) demonstrated that feature-tracking algorithms could identify colon flexures with 86% accuracy. 39 Koulaouzidis et al. (2018) advanced 3D reconstruction techniques that assist in contextualizing capsule location using Simultaneous Localization and Mapping principles. 40

These technologies, if implemented clinically, can reduce reader variability and improve lesion localization. They also support more accurate triage to colonoscopy, guiding the endoscopist to the correct segment and minimizing patient discomfort and repeat examination time. In future iterations, AI could integrate with full capsule path reconstruction platforms, producing a spatial map of mucosal visualization and landmark traversal.

## DOMAIN. Completeness of procedure

### AI Task 2.3—Correct assignment of Pathology to colonic Segments

The framework for AI-assisted assignment of pathology to colonic segments is summarized in Table 2.3 **.**

**Table 2.3:** Correct assignment of pathology to colonic segments.

Technique	CCE
Domain	Completeness of procedure
AI task	Identification of colonic landmarks and segments; assignment of pathology to colonic segments
Description	Real-time AI-assisted CCE reader assessment of colonic landmarks (appendiceal orifice, ileocecal valve, hepatic and splenic flexure, hemorrhoidal plexus, and segment identification [cecum, ascending colon, transverse colon, left colon, rectum], and real-time assignment of pathology to colonic segments)
Performance measure	Correct assignment of pathology to colonic segments
Rationale	Recognition of colon segments is essential for assigning accurate segmental cleansing scores, determining lesion location, and informing clinical decision-making. While expert readers rely on visual cues and experience to delineate anatomical transitions, interobserver variability remains high. AI offers a standardized, potentially superior method to assist in segment identification. Enhanced localization enables accurate assessment of mucosal visualization per segment and optimizes lesion retrieval during subsequent procedures. AI models should integrate image data and auxiliary localization signals (e.g., signal strength or trajectory estimation) to increase robustness in segmental classification Accurate localization of detected pathology is essential for effective clinical management. Segment-level assignment of lesions informs polypectomy planning, surveillance, and therapeutic decisions. Mislocalization can lead to incorrect follow-up strategies or repeat procedures. While some capsule platforms previously featured trajectory mapping, newer systems prioritize intraluminal imaging. AI has the potential to bridge this gap and enhance localization despite the absence of visual trajectory cues.
**Reference Standard**	Identification of colon segments by experienced readers and/or signal-based 2D or 3D capsule trace reconstructions, with correct assignment of pathology to colon segments
**Expected value**	Minimum expected value: ≥90% concordance with expert-reader consensus across the full colon. Aspirational value: ≥95% concordance with validated reference standards, where available.

**STATEMENT**

**For acceptance of AI in the identification of colon segments and the correct assignment of pathology, AI systems should demonstrate consistency and accuracy comparable with those of experienced CCE readers.**

Agreement: 91.0%, at the second voting round.

## Discussion

Lesion retrieval at subsequent colonoscopy is facilitated by accurate segmental localization during CCE. Matching of polyps between CCE and colonoscopy has also been based on segmental localization in addition to size and morphology. 41 Additionally, the extent of inflammatory diseases can be assessed noninvasively, particularly when histology has been obtained beforehand.

Studies have applied a convolutional neural network (CNN) in small-bowel capsule endoscopy (SBCE) for the identification of segments of the GI tract, such as the stomach, SB, and colon. As SB CE usually provides no complete colon visualization, colonic segments were not differentiated. 42 43 44 45

Recently, a CNN and a long short-term memory network showed that the mean time differences between the model predictions and ground truth were 4.3 ± 9.7 min for the stomach and 24.7 ± 33.8 min for the small intestine, 43 which seems quite high for these more easily identifiable segments. No colon segments were assessed.

Identification of the transition from the ileum to the colon and passage from the rectum outside the body is facilitated by several visual clues. However, identification of colonic segments by landmarks proved to be difficult for expert clinicians due to a paucity of definite morphologic features of the different segments. 46 This constraint is also limiting the training of AI. Feature Point Tracking-Based Localization retrospectively had an overall accuracy of 86%, which was decreasing from distal to proximal colonic segments. 39 This method was also suggested for application as a localization tool in future AI algorithms, guiding the entire CCE flow. 47

Future research might focus on training AI in the assessment of the colonic segment, not only based on luminal images but also on additional data, for example, provided by localization based on signal strength 48 49 externally actuated capsule passage, and others. Availability of time stamps from the recorded CCE videos is considered crucial for analysis.

Polyp localization in studies on AI assistance in CCE is often referred to as the localization of the polyp within a still image frame, but not to its localization about colon segments, 50 which is the focus here.

Baltes et al. 51 reported polyp localization for the rectum, sigmoid, descending, transverse, and ascending colon. This was possible by additionally applying localization trace, based on signal analysis as provided by PillCam Colon2 and RAPID 8 software (Medtronic, Dublin, Ireland). No AI assistance was used.

The graphical display of the capsule path had been abandoned in later software versions. Hence, later studies used fewer segments, for example, as the ScotCap study with right colon, transverse colon, and left colon (including rectum), 14 while Hotta et al. described the rectum separately. 52

Sakai et al. 53 divided the colon into the right and left colon only. Nonetheless, this study involved patients who had undergone CRC surgery, typically leaving only one hemicolon remaining. These studies did not apply AI either.

In general, AI training and testing for assessment of disease extent in certain colon segments are hampered by insufficient data on ground truth due to poor interobserver agreement on segment-defining landmarks using software without a graphical display of capsule path. 46 Currently, AI models cannot replace this missing graphical information by using input from endoscopic intraluminal images alone. Further efforts are needed to make reliable AI-generated information on colon segments available. In addition to localizing polyps to subsegments of the colon, this could be especially helpful for assessing the extent of inflammatory lesions in the colon, for example, in ulcerative colitis.

A study applied AI assistance in localizing inflammatory lesions to the segments of either the SB or the colon. 54

Higuchi et al. applied several CNNs to classify the severity of ulcerative colitis for different colonic segments. However, these segments were manually defined from the PillCam Colon videos. 55

## DOMAIN. Completeness of procedure

### AI Task 2.4—CCE Path Reconstruction

The proposed framework for AI-assisted CCE path reconstruction is summarized in Table 2.4 **.**

**Table 2.4:** CCE path reconstruction.

Technique	CCE.
Domain	Completeness of procedure.
AI task	Real-time AI-assisted CCE path reconstruction.
Description	CCE path reconstruction.
Performance measure	Proportion of correct CCE path reconstruction by AI assistance.
Rationale	CCE path reconstruction can assist in the localization and matching of pathology, in the assessment of regional transit times and in the cleanliness of the colon. Determining the location of a detected polyp supports appropriate follow-up—e.g., flexible sigmoidoscopy versus full colonoscopy—and guides polypectomy during therapeutic endoscopy. Reliable segmental assignment also enables differentiation between unique lesions and repeated frames of the same lesion. Reconstruction of the capsule path, historically facilitated by software like RAPID 8, enhances segment identification but is no longer standard. AI could bridge this gap using visual and temporal features from video data.
Reference Standard	Path reconstruction by experienced CCE readers, including graphical trajectory outputs when available.
Expected value	Minimum expected value: ≥80% agreement with expert localization. Aspirational value: ≥90% agreement, with a documented confidence score for each prediction. The confidence score should be interpreted as a probability or uncertainty estimate and does not alter the agreement thresholds.

**STATEMENTS**

**For the acceptance of AI in the assessment of CCE path reconstruction, its performance should be comparable to that of experienced CCE readers**

Agreement: 91.7%, at the first voting round.

**AI-driven path reconstruction must produce interpretable outputs, showing the capsule’s journey through the colon.**

Agreement: 95.8%, at the first voting round.

## Discussion

The localization of polyps within the CCE path is an important criterion for matching polyps, specifically for differentiating between images of various polyps and images of the same polyp. Besides localization and matching pathology, CCE path reconstruction can help assess regional transit times and regional cleanliness.

Radiofrequency signal analysis has been used to create a graphical path for SB CE. This graphical display of the capsule’s trajectory has been validated through intermittent radiographs. The accuracy of PillCam SB (Medtronic, Dublin, Ireland) 2D localization compared with radiographs at identical time points was 37.7 mm. 56 Similarly, radiographic validation of the radiofrequency-based Capsule 3D Track function of the Olympus EC-10 EndoCapsule system demonstrated mean localization errors of 2.00 cm, 2.64 cm, and 2.51 cm along the X, Y, and Z axes, respectively; the authors reported a mean total spatial error of 13.26 cm³ (SD 22.72). 49

PillCam Colon2 with RAPID 8 software similarly provided a graphical path illustration, enhancing identification of colon segments. 51 The localization feature is currently unavailable and has not been included in subsequent software versions.

The ability of AI to reconstruct the CCE path solely from endoluminal images and videos presently seems limited. Missing external localization limits both AI output and the assessment of ground truth, as shown by the high interobserver variance among experts in identifying colonic landmarks. 46 Accordingly, agreement between AI models and experts in predicting colonic segments is currently only moderate. 39 57

In this context, a confidence score refers to an interpretable probability or uncertainty estimate attached to each predicted segment or reconstructed path. It does not modify the minimum acceptable or aspirational value thresholds; rather, it should help clinicians identify predictions that require manual verification.

## DOMAIN. Completeness of procedure

### AI Task 2.5—Visualization of SB Mucosa

The proposed framework for AI-assisted SB mucosal visualization is summarized in Table 2.5 .

**Table 2.5:** Visualization of the small bowel mucosa.

Technique	CCE.
Domain	Completeness of Procedure.
AI task	Assessment of complete small-bowel visualization.
Description	AI-assisted assessment of completeness of small bowel mucosal visualization.
Performance measure	Accuracy of complete small bowel visualization determination compared with expert assessment and procedural landmarks.
Rationale	Complete small bowel visualization is essential for reliable detection of pathology, particularly in cases of obscure GI bleeding, suspected Crohn’s disease, or small bowel tumors. In SBCE, complete visualization is defined by the identification of both the pylorus and cecum. AI could assist by identifying anatomical landmarks, classifying mucosal visibility, and quantifying coverage across time.
Reference Standard	Performance by experts in confirming complete small bowel visualization based on procedural documentation and landmark identification.
Expected value	Minimum expected value: Accurate detection of complete small bowel visualization in ≥85% of procedures, with correct identification of both pylorus and cecum. Aspirational value: ≥95% accuracy in visualization classification and anatomical landmark recognition, with quantification of mucosal visualization quality and completeness.

**STATEMENT**

**To be accepted for clinical use, AI systems assessing the completeness of small bowel visualization must demonstrate performance comparable to expert reviewers, based on recognized anatomical landmarks and visualization quality indicators.**

Agreement: 100.0%, at the first voting round.

## Discussion

Visualization of the SB is optional for a CCE *sensu stricto* , although it adds valuable information, and is mandatory in pan-enteric CE *.* While the cleanliness of SB during CCE seems to be improved by the administered booster solutions, the percentage of visualized SB footage during CCE may vary. Documentation of capsule passage from the terminal ileum into the cecum is relevant in assessing completeness of CCE.

Adequate SB mucosal visualization is a key performance indicator 9 that should be obtained in >80% of the examinations. Although several scales have been proposed over the years, 58 only a few have been validated, and they are infrequently used, albeit recommended, 59 in everyday clinical practice, due to time-consuming, often subjective processes. The rationale for AI-assisted algorithms for assessing SB cleansing is to provide an objective standardization of these processes, with decreased interobserver variability. Computerized assessments with color-intensity-based approaches have shown promising results, either when performed on the color bar 60 61 62 63 or directly on CE still images, 64 65 66 although these approaches are limited by the difficulty of distinguishing color variations due to obscuration and lesion-associated color variations, such as bleeding. High accuracy compared with expert CE readers was also achieved with deep learning and CNN algorithms, albeit their clinical application remains limited to single-frame analyses. 28 67 68 69 70 Furthermore, a whole-video automated cleanliness assessment appears technically feasible, 71 72 even when the videos are obtained from different sources. 73 An AI software (AXARO.AI, Augmented Endoscopy, Paris, France) performs automated reading of endoscopic video capsule images, detects abnormalities, and indicates whether the quality of the intestinal preparation is satisfactory (per quartile and total). The methods initially used for small intestine investigations are now being extended to the colon. The software is now CE-marked and commercially available online. 74

Procedural completeness (i.e., cecum/stoma landmarks) is a key performance indicator for CE procedures, 9 as misinterpretation of capsule passage may result in missing pathology and inaccurate SB transit times (thereby affecting the potential yield of subsequent endoscopic interventions). As the identification of landmarks is a subjective task that may be burdened by time-consuming processes, especially in cases of poor bowel preparation, several papers have investigated the reliability of computed algorithms to provide endoscopists with automated systems to accomplish this evaluation. 38 42 45 Although these systems, based on deep learning and CNN technologies, exhibit promising performance results in organ recognition, data from a recent real-life study with proprietary software still show a wide discrepancy between AI-assisted and standard reading in recognizing transitional areas. 75

## DOMAIN. Identification of Pathology

### AI Task 3.1—Reading Time

The expected impact of AI on CCE reading time is summarized in Table 3.1 .

**Table 3.1:** Reading time.

Technique	CCE.
Domain	Identification of pathology.
AI task	Automated reading.
Description	AI-assisted reading of CCE.
Performance measure	Reading time.
Rationale	CCE reading is time-intensive and subject to reader fatigue, compromising diagnostic accuracy. AI can pre-screen frames and flag suspected pathology, drastically reducing human reading time while maintaining or improving lesion detection. This has been demonstrated in SB CE and is increasingly validated for colon applications. AI tools should include frame selection, lesion prioritization, and interpretable outputs to support rapid and confident review by clinicians.
Reference Standard	Conventional reading time.
Expected value	Minimum expected value: ≥50% reduction in reading time compared with human experts. Aspirational value: ≥85% reduction without loss of sensitivity or specificity.

**STATEMENT**

**For the acceptance of AI in automated CCE reading, AI systems should reduce reading time by ≥50% while maintaining noninferior lesion detection (statistical margin 5%, absolute value).**

Agreement: 95.8% at the first voting round.

## Discussion

Analysis of a CCE video is a time-consuming process that requires prolonged attention and focus, making it a major burden for the reader. Experience from SBCE has shown that the reader’s accuracy declines progressively after reading just one capsule study, 76 and a similar effect is expected in CCE.

Reducing reading time is one of the benefits expected from applying AI in CCE evaluation. Several studies have shown that AI systems can process images much faster than human readers, with high-performance for the automated detection of specific lesions: a deep learning system developed by a Portuguese group has performed with an approximated reading rate of 65 frames per second, which translates to a 13-minute review of a full-length CCE video containing an estimated 50,000 frames. 77 78 This reading time reduction may also be maintained when comparing conventional human reading to AI-assisted reading with a dedicated software for automated detection of colonic polyps: the results of a single-center study showed that the AI software application reduced the time needed to reach the same accuracy as human reading by a factor of 6 (47 vs. 8 min). 79 In a pan-enteric CE (PCE) study that included 131 patients with a median recording time of 303 min, the AI tool’s pooled median review time was 3.2 min per patient while maintaining high diagnostic accuracy to rule out IBD in patients suspected of Crohn’s disease. 80

Furthermore, a multicenter combined prospective and retrospective study (CESCAIL) aims to determine the performance measures (including reading reduction) of a CNN-based AI-enabled analysis tool for polyp detection, compared with the “gold standard” conventional human reading—the results are pending because recruitment is ongoing. 81

## DOMAIN. Identification of Pathology

### AI Task 3.2—Polyp Matching to Avoid Double Reporting

The proposed framework for AI-assisted polyp matching is summarized in Table 3.2 .

**Table 3.2:** Polyp matching within the same CCE video to avoid duplicating reporting.

Technique	CCE
Domain	Identification of pathology
AI task	Identified polyps must be carefully matched and verified to prevent duplication of the same pathology and ensure accurate reporting
Description	AI-assisted detection can help avoid duplicating the reporting of the same polyp or other lesions in CCE, which may otherwise raise concerns and cause confusion during subsequent colonoscopies. This could potentially lead to unnecessary follow-up endoscopies
Performance measure	The accuracy of predicting the number of polyps within the same CCE video using an expert panel as ground truth or comparing to subsequent conventional endoscopy in experienced bowel cancer screeners is limited due to the higher miss rate observed among general endoscopists
Rationale	To develop a high-performing model to accurately detect polyps within the same CCE video, preventing double-counting and reducing unnecessary procedures. By eliminating the risk of overestimated polyp numbers due to duplicate reporting, the model will also alleviate patient anxiety by reducing discrepancies between CCE and subsequent colonoscopy results. Additionally, it will streamline the CCE reading process, allowing clinicians to spend less time reviewing the entire video to determine whether polyps seen at different angles are, in fact, the same. This tool will assist clinicians in making more accurate assessments of similar polyps. As localization is not always precise in CCE, the model could also improve capsule tracking, helping clinicians avoid confusion when the capsule travels backward or revisits previous segments, reducing the risk of mistaking a single polyp for multiple ones, with the added benefit of improving capsule localization when the same polyp is seen later in the video. AI output should include frame-level matches and confidence scores indicating which polyp appearances have been classified as duplicates
Reference Standard	Expert CCE-reader consensus using polyp matching criteria from rationalizing polyp matching criteria in colon capsule endoscopy: an international expert consensus through RAND (modified Delphi) process. 56
Expected value	AI-assisted polyp matching within the same CCE video should be at least comparable to, and ideally superior to, experienced CCE readers or expert CCE-reader consensus

**STATEMENT**

**AI-assisted polyp matching within the same CCE video should be at least comparable with an experienced CCE reader and ideally superior.**

Agreement: 83.3%, at the first voting round.

## Discussion

The aim is to develop a high-performing model that accurately identifies and compares polyps within the same CCE video to prevent double counting and reduce unnecessary procedures. Duplicate reporting of polyps can increase polyp counts, leading to unwarranted follow-up procedures and unnecessary patient anxiety when discrepancies arise between CCE and subsequent colonoscopy results. 56 The current method requires clinicians to spend significant time reviewing entire video segments to determine whether polyps seen at different angles are the same, contributing to inefficient clinical workflows. AI and ML could streamline this process, reducing the time clinicians spend analyzing videos and improving the accuracy of polyp assessments. Additionally, this tool will help address challenges with capsule localization, as clinicians can sometimes lose track of the capsule’s movement, mistaking a single polyp for multiple ones when revisiting prior segments. This model could also provide a secondary benefit by improving capsule localization if the same polyp is seen later in the CCE video. A more efficient and accurate system would ultimately improve clinical outcomes, reduce patient anxiety, and optimize the use of CCE in clinical practice.

In the available literature, no AI tools have been identified in the research, commercial, or clinical sectors for this purpose. However, previous large-scale studies, such as Blanes-Vidal et al. (2018), as well as studies like ScotCap and the NHS England First Study, may offer valuable data for model training. 14 82 83 These studies could leverage CCE video data to build and train a robust image recognition model.

## DOMAIN. Identification of Pathology

### AI Task 3.3— Polyp Size Measurement

The expected standards for AI-assisted polyp size measurement are summarized in Table 3.3 .

**Table 3.3:** Accurate polyp size measurement.

Technique	CCE.
Domain	Identification of pathology.
AI task	Polyp size measurement.
Description	AI-assisted size measurement of colorectal polyps.
Performance measure	Accuracy of polyp size measurement compared with expert assessment and colonoscopy/histological reference, where available.
Rationale	Polyp size is a critical determinant in surveillance intervals and clinical risk assessment. Polyps ≥10mm typically require endoscopic resection and may carry increased malignant potential. In CCE, estimating size is inherently limited because there is no reference scale or comparison tool. AI could support objective estimation by analyzing image features, perspective cues, and comparative frame data. AI outputs should include interpretable size overlays or visual indicators and align with clinical thresholds for resection and surveillance.
Reference Standard	Histology.
Expected value	Minimum expected value: ±2 mm size estimation accuracy in ≥80% of cases. Aspirational value: ±2 mm accuracy in ≥90% of cases, with correlation to reference measurements where available.

**STATEMENT**

**For the acceptance of AI in polyp size estimation, systems should demonstrate size measurement accuracy within ±2 mm in ≥80% of cases.**

Agreement: 95.8%, at the first voting round.

## Discussion

The size of polyps is crucial in assessing the risk of CRC and in guiding clinical management. Larger polyps, especially those measuring ≥10 mm, are linked to advanced neoplasia and influence the recommended surveillance intervals according to established guidelines. However, accurate polyp sizing remains challenging in CCE because no fixed intraluminal reference object is available, the capsule-to-lesion distance and viewing angle are uncontrolled, and the absence of insufflation results in variable luminal distension, potentially altering the apparent size and morphology of lesions compared with conventional colonoscopy. Thus, the 10 mm threshold in colonoscopy may not apply in CCE.

Currently, size estimation in CCE is largely based on reader experience and visual interpretation, introducing interobserver variability. 41 84 AI offers a potential solution by providing automated, reproducible measurements derived from spatial and geometric cues present within the image data. CNNs and other computer vision techniques can be trained to assess polyp diameter using annotated datasets where lesion dimensions are confirmed by colonoscopy or histopathology.

Several pilot studies have suggested promising accuracy using deep learning-based methods on datasets derived from colonoscopy. 85 86 87 Techniques such as scale-invariant feature mapping, shadow depth estimation, or cross-frame triangulation are under investigation. Most models are still in development, with validation limited to retrospective datasets.

Despite recent advancements, there are still technical limitations to consider. These include image distortion caused by lens curvature, motion blur, and inconsistencies in lighting. It is important to address these factors during the training and validation phases. Furthermore, the AI system must be capable of distinguishing between full views and partial views of polyps to prevent both overestimation and underestimation.

Clinical implementation would benefit from models that output not only a single size estimate but also confidence intervals, visual overlays, and a qualitative size category (e.g., <6, 6–9, ≥10 mm). This information should be integrated into the diagnostic report and guide downstream decision-making. Overlays should visually mark measurement reference points on the lesion to support interpretability.

It is also important that AI models be tested across varied patient populations, bowel preparation levels, and capsule platforms. External validation using colonoscopy and/or surgical pathology as the reference standard remains the gold standard.

Ultimately, AI-driven size measurement has the potential to reduce variability and improve the reliability of CCE in clinical workflows. However, further prospective validation is needed before widespread adoption.

## DOMAIN. Identification of Pathology

### AI Task 3.4—Sensitivity and Specificity for CRC and Polyps

The proposed sensitivity and specificity thresholds for AI-assisted CRC and polyp detection are summarized in Table 3.4 .

**Table 3.4:** AI-assisted CCE Sensitivity and specificity for detection of CRC and polyps.

Technique	CCE.
Domain	Identification of pathology.
AI task	Detection/Identification of CRC and polyps.
Description	Automated detection of colorectal neoplasia, including the sensitivity and the specificity of: CRC. Polyps ≥ 6 mm. Polyps ≥ 10 mm. Polyps overall.
Performance measure	CRC and polyp detection rate.
Rationale	Accurate detection of significant lesions can help triage patients and increase screening uptake. AI-assisted polyp detection with CCE must be validated on diverse datasets and benchmarked against histologically confirmed neoplasia.
Reference Standard	Conventional colonoscopy with histological confirmation.
Expected value	Minimum expected value for CRC and for polyps ≥6mm: Sensitivity ≥90%; Specificity ≥90%. Aspirational for CRC and all polyp sizes: Sensitivity ≥95%; Specificity ≥95%.

**STATEMENT**

**For acceptance of AI in polyp and CRC detection via CCE, AI systems should demonstrate sensitivity and specificity of at least 90% in detecting polyps measuring ≥6 mm**

Agreement: 83.3%, at the first voting round.

## Discussion

A meta-analysis reported that CCE had a per-patient sensitivity of 71% for detecting polyps of any size and 68% for significant findings, defined as polyps ≥6 mm and/or three or more lesions. These results compare favorably with other noninvasive or less-invasive options for CRC screening. Furthermore, with flexible spectral imaging color enhancement, polyp detection is enhanced, allowing for differentiation between adenomatous and hyperplastic polyps (sensitivity of 91.2% and accuracy of 88.2%). 88 89 A challenge is the detection of sessile serrated lesions (SSLs), which account for 15–30% of CRCs and an even higher proportion of interval cancers, as they are often missed during colonoscopy. 90 Unfortunately, the scenario is not significantly better for CCE, with sensitivities of 29% and 33% for SSLs >6mm and >10mm, respectively. 91

The introduction of AI in the field of CRC screening began with colonoscopy, for which numerous studies have been conducted to date. Notably, GI Genius (Medtronic, Dublin, Ireland), the first FDA-approved AI system to aid polyp detection during colonoscopy, has demonstrated improved polyp detection rates (from 40.3% to 54.8%) compared to conventional colonoscopy without AI. 92 Although this increase is primarily attributable to smaller polyps, the detection of all neoplasia types also improved. In a prospective colonoscopy study, computer-aided detection/diagnosis systems were also shown to directly reduce the number of missed adenomas, suggesting that they could help lower the rate of interval cancers. 93

Current studies focus on the diagnostic accuracy and efficiency of AI-assisted CCE in identifying colonic lesions, such as polyps, which are precursors to CRC. Two studies are highlighted for their focus on detecting polyps and cancers. First, Yamada et al. studied the implementation of AI algorithms for CRC detection in the context of video frames of colorectal neoplasms, such as polyps and cancers extracted from CCE exams. A total of 17,783 frames from 178 patients were analyzed. The algorithm showed promising results, including an area under the receiver operating characteristic curve (AUROC) of 0.90, sensitivity of 79%, and specificity of 87% for detecting polyps and CRC. 94 Subsequently, a multicenter retrospective study conducted by Mascarenhas et al. focused on developing a CNN-based algorithm for the automatic detection of protruding lesions in the colon during CCE, including epithelial tumors, subepithelial lesions, and polyps. A total of 5,715 frames from 124 patients were analyzed. This model demonstrated a sensitivity of 90%, specificity of 99.1%, PPV of 98.6%, and NPV of 93.2% in detecting protruding lesions. 77

Blanes-Vidal et al. developed a deep CNN for autonomous detection and localization of colorectal polyps in CCE. This study involved 255 patients who tested positive for a FIT as part of Denmark’s national screening program. The researchers analyzed 375 polyps detected through CCE and compared them to findings from conventional colonoscopy to evaluate the effectiveness of their CNN-based detection system. Using a polyp matching algorithm, they achieved a high matching rate of 87%, indicating that CCE can reliably detect polyps and align well with colonoscopy findings. However, a notable limitation was the tendency of CCE to overestimate polyp size compared to colonoscopy and histopathology. Nevertheless, the autonomous detection algorithm demonstrated exceptional performance, achieving an accuracy of 96.4%, sensitivity of 97.1%, and specificity of 93.3%, surpassing previous methods. 41

The same group subsequently conducted a substudy using a modified ZFNet architecture, which was trained on 11,300 CCE images and evaluated on an independent test set of 1,695 previously unseen images. The model achieved an accuracy of 98.0%, a sensitivity of 98.1%, and a specificity of 96.3%. The CNN was further integrated into a Faster R-CNN framework that enabled polyp localization with sensitivity and specificity of 95.3% and 92.8%. Saliency maps revealed the network’s focus on edge features of polyps, enhancing its interpretability and potential utility in clinical settings. 95

Gilabert et al. introduced the AI-Tool, a CNN-based system designed to enhance colonic polyp detection in CCE videos. Compared to the traditional RAPID Reader Software v9.0, the AI tool improved overall Se from 81.1% to 87.8%. For small polyps (<6 mm), sensitivity increased from 76.9% to 85.4%, while for large polyps (≥6mm), it rose from 85.7% to 91.2%. The sensitivity for low-visibility polyps saw the most significant improvement, from 58.3% to 80.0%. The system also reduced screening time sixfold, from 47 to 8 minutes, by prioritizing frames with high polyp probabilities and generating heatmaps to guide reviewers. 79 Bridging the gap between automated analysis and human oversight, saliency and heatmaps improve transparency and facilitate the integration of AI systems into clinical workflows, paving the way for more reliable and efficient diagnostic processes. 41 79

Mascarenhas et al. were also pioneers in the development of AI technology applied to CCE. In 2021, they developed a CNN using 3,640 images, capable of detecting protruding lesions with Se, Sp, positive, and negative predictive values of 90.7%, 92.6%, 79.2%, and 96.9%, respectively. The AUROC for detecting protruding lesions was 0.97. 96 In 2022, this research group published a retrospective multicenter study that resulted in the development of a CNN model. This model is designed for the automatic detection and differentiation of various colonic mucosal lesions, including erosions, ulcers, vascular lesions, and protruding lesions, as well as the detection of blood in CCE images. The network accurately detected 89.1% of protruding lesions (including polyps, epithelial tumors, submucosal tumors, and nodules) with minimal false negatives. Although specificity for protruding lesions was not provided exclusively, it was inferred to be high, as the overall specificity for mucosal lesions reached 98.5%. 78

A key limitation is that several CCE AI studies have been developed or tested on selected still images or extracted frames rather than unselected full-length videos. As in colonoscopy AI research, frame-based performance may overestimate real-world accuracy because full-video studies introduce motion blur, residual stool, partial lesion views, repeated appearances of the same lesion, variable bowel preparation, and workflow constraints. Therefore, the proposed detection thresholds should be validated in prospective, externally validated, full-video CCE datasets before being considered implementation standards.

Similarly, false negatives occur mainly due to low contrast between polyps and surrounding tissue, partialness (lesion partially visible), small-sized lesions, and suboptimal image quality, mainly due to poor bowel preparation. 78 97 Finally, perhaps the most crucial factor for misclassification is the lack of consistent morphology, size, texture, and color features among polyps, even within the same patient. 78 79 98

To optimize the clinical use of CCE, high sensitivity must be complemented by addressing sources of false positives and negatives. Improvements in bowel preparation, enhanced image quality, and refinement of AI training datasets are critical steps toward minimizing errors and maximizing diagnostic reliability.

## DOMAIN. Identification of Pathology

### AI Task 3.5—AI-assisted CCE false positive rate

The proposed thresholds for AI-assisted false positive rates are summarized in Table 3.5 **.**

**Table 3.5:** AI-assisted CCE false-positive rate.

Technique	CCE.
Domain	Identification of Pathology.
AI Task	False Positive Rate.
Description	AI algorithms used in CCE may flag numerous potential abnormalities during review. This task aims to quantify and minimize the false positive rate of AI systems, thereby reducing unnecessary human workload and minimizing follow-up procedures that arise from incorrect AI alerts.
Performance measure	Rate of false positive findings (e.g., number of false-positive findings per complete CCE examination); reduction in unnecessary reader verification and follow-up procedures.
Rationale	While AI significantly enhances lesion detection, high false positive rates may undermine clinical utility by increasing review burden, causing reader fatigue, and potentially prompting unnecessary diagnostic procedures. An acceptable false positive rate is essential to ensure AI improves, rather than hinders, workflow efficiency.
Reference Standard	Expert human reader consensus or adjudicated ground truth based on histopathology, endoscopic confirmation, or validated dataset annotations.
Expected value	Minimum expected value: false positive rate does not significantly exceed that of expert readers. Aspirational value: <5 false positives per complete CCE study in well-annotated datasets; this is a consensus-derived expected-value benchmark requiring prospective validation.

**STATEMENT**

**For acceptance of AI in CCE pathology identification, the system must demonstrate a false positive rate not exceeding that of experienced human readers and ideally maintain <5 false positives per complete study in well-annotated datasets.**

Agreement: 83.3%, at the first voting round.

## Discussion

The integration of AI into CE, particularly CCE, has ushered in a new era of diagnostic efficiency and Se. By leveraging CNNs and other ML architectures, AI systems have demonstrated substantial capability in detecting clinically relevant lesions such as polyps, bleeding, and ulcers. 99 However, one of the critical challenges in implementing these technologies in routine clinical practice lies in the control of false positive rates. A high FPR can undermine the benefits of automation by increasing the cognitive burden on clinicians, triggering unnecessary follow-up investigations, and diluting clinical confidence in AI-assisted outputs. Therefore, minimizing FPR is essential for both the clinical viability and scalability of AI-assisted CCE systems. 100

False positives in CCE occur when AI algorithms incorrectly identify normal or nonsignificant findings as pathological. These misclassifications can stem from several sources, including poor bowel preparation, lighting artifacts, residual debris, mucosal folds, or normal anatomic variants. 101 While human readers typically have the interpretive experience to discount such findings, AI systems trained on imperfect or limited datasets may be prone to overflagging, especially when prioritizing sensitivity. 102 This trade-off between Se and Sp is well recognized in AI diagnostics. However, in CCE, where thousands of frames are generated per study, even a modest increase in false positives per study can translate into significant additional review time and clinician fatigue. 103 The impact of false positives is not trivial. From a workflow standpoint, clinicians must manually verify each flagged frame, often reviewing numerous irrelevant images that do not contribute to clinical decision-making. This erodes one of AI’s primary advantages: reducing the time burden associated with manual video review. 104 In some instances, high false positive rates can negate the perceived efficiency gains of AI, making technology less appealing to end-users. Moreover, frequent false alerts can foster “alarm fatigue,” leading clinicians to become desensitized to AI prompts and increasing the risk of overlooking true positives. 105 At a systemic level, false positives can lead to unnecessary diagnostic colonoscopies, imaging studies, or even interventions, all of which increase patient burden, healthcare costs, and resource utilization. 6

To address this, the design of AI systems must include rigorous optimization of the balance between detection Se and Sp. Several strategies are being explored to reduce false positive rates without compromising diagnostic accuracy. These include training on larger and more diverse datasets, improving annotation quality, and incorporating ensemble or hybrid models that combine frame-level and sequence-level assessments. 106 Attention mechanisms and temporal smoothing can also help the system contextualize findings, reducing overinterpretation of isolated frames. 100 Furthermore, the evaluation of FPRs must be done in a clinically meaningful way. It is not sufficient to report per-frame metrics; rather, per-study false positive counts and their practical implications (e.g., time spent reviewing false flags, downstream investigations triggered) must be considered. In the absence of robust CCE-specific evidence, the proposed target of fewer than five false positives per complete CCE study should be interpreted as a consensus-derived aspirational value, not as a validated clinical threshold. It will require prospective validation in well-annotated full-video datasets before it can be used as an implementation standard.

Another important consideration is the reference standard against which false positives are adjudicated. Ideally, expert consensus with access to histopathological confirmation or follow-up endoscopy should be used to distinguish false from true positives. 107 In the absence of this, annotated benchmark datasets validated across multiple centers and populations are essential to ensure algorithm generalizability and to avoid overfitting narrow image phenotypes. The regulatory and ethical dimensions also warrant discussion. From a governance perspective, high FPRs can pose medicolegal challenges, particularly if they lead to unnecessary procedures. 108 There is also a risk of clinician overreliance on AI outputs, potentially leading to diminished vigilance. As such, clinical deployment must be accompanied by training protocols that emphasize AI’s role as an adjunct, not a replacement, for expert interpretation. Transparency of the AI’s decision logic, along with confidence scores and explainability features, can further help clinicians calibrate their trust in the system and respond appropriately to flagged images. 109

## DOMAIN. Identification of Pathology

### AI Task 3.6—AI-assisted CCE nonneoplastic pathology detection rate

The proposed framework for AI-assisted detection of nonneoplastic pathology is summarized in Table 3.6 .

**Table 3.6:** AI Task—AI-assisted CCE nonneoplastic pathology detection rate.

Technique	CCE.
Domain	Identification of pathology.
AI task	Nonneoplastic pathology detection rate.
Description	AI-assisted detection of nonneoplastic pathologies, focusing on conditions such as colitis, vascular lesions, and other abnormalities, through automated analysis of colon visualization and classification.
Performance measure	Detection rate of nonneoplastic colonic findings, including signs of inflammation, erosions, ulcerations, signs of bleeding, and vascular lesions.
Rationale	Assess the ability of the AI model to correctly identify pathological findings compared to human readers or traditional methods (e.g., optical colonoscopy). This includes metrics such as Se, Sp, and predictive values. Increase the detection of relevant findings, such as erosions, ulcerations, vascular lesions, and signs of bleeding, compared to manual review. Minimize the number of clinically significant findings missed during initial evaluations, especially in challenging images or suboptimal bowel preparation.
Reference Standard	Expert human-reader consensus, adjudicated ground truth based on endoscopic confirmation, or validated dataset annotations.
Expected value	The expected values include higher diagnostic accuracy, improved diagnostic yield, and a significant reduction in missed lesions for clinically relevant findings with AI assistance.

**STATEMENT**

**For acceptance of AI in nonneoplastic pathology detection, systems must demonstrate diagnostic accuracy, diagnostic yield, and a rate of missed lesions equivalent to that of conventional CCE interpretation, with performance equal to or exceeding expert human readers and optical colonoscopy standards.**

Agreement: 100.0%, at the second voting round.

## Discussion

### Diagnostic Accuracy

The efforts in the field of AI in CCE have primarily focused on detecting neoplastic pathologies such as cancers and polyps. 110 111 However, there is now a growing recognition of the need for AI-supported detection of nonmalignant lesions. This shift in focus aligns with developments in AI tools for SBCE, where the prevalence of malignant findings is low, and the primary objectives include identifying inflammatory lesions and sources of occult bleeding. As a result, similar advancements in CCE are now being explored. AI models have been developed for the detection of nonneoplastic pathology, specifically in CCE, and the research in this field is expanding continuously.

### Inflammation

With the introduction of the PCE approach, especially targeting patients with established or suspected inflammatory bowel disease, AI models for the detection of inflammation in the colon are presented. These models have the potential to point out subtle patterns and anomalies that are at risk of being missed by the human eye. 110 Inflammatory signs can be difficult to both detect and classify correctly, and AI could therefore be a great asset to the accuracy of reporting. Cambay et al. present an algorithm for the detection and classification of different findings in CE. In a mixed dataset including 200 images depicting ulcerative colitis, the algorithm correctly identified and classified 196 of these images. 112 Other groups have developed AI models for the detection of colonic ulcers and erosions, Ribeiro et al. showing a sensitivity, specificity, and overall accuracy of 96.9%, 99.9%, and 99.6%, 113 while Kratter et al. report an accuracy reaching 93.7%. 114 Majtner et al. present a deep learning model for the detection and classification of Crohn’s disease lesions in PCEs. 54 80 When looking at the results for the colonic lesions, they report a Se, Sp, and overall accuracy of 94.2%, 100%, and 98.1%.

In a similar study, the reported Se and Sp of the AI-assisted observer in PCE were similar to those of the conventional observer, while the review time was only 3.2 min with the AI tool. 80 Overall, the AI-reported detection of various signs of inflammation in CCE is very promising.

Aside from detecting images of inflammation in the colon, AI is utilized in the grading of inflammation severity. 55 115 A CNN-based automated scoring system for grading the severity of inflammation in the small and large bowel, respectively, has been presented. 115 The study found a strong correlation between the AI and expert-reported score for the SB, but no correlation for colonic evaluations.

### Vascular Lesions and Blood

Mascarenhas et al. present two articles reporting on AI models for the identification of blood in the colon with a Se, Sp, and overall accuracy of 99.5–99.8%, 93.2–99.8%, and 96.6%. 78 116 One of these studies further reports a detection rate of vascular lesions by the CNN model of 85.7% (30 of 35 lesions). 78 Furthermore, a study reporting on PCEs shows promising results with a Se, Sp, and overall accuracy of 86.4%, 98.3%, and 95.0%. 117 The main reason for misclassifications is reported to be the presence of bubbles or fecal matter obscuring the view.

### Limitations and Challenges

Despite these advances, some challenges remain for CCE to effectively and maximally implement and optimize AI. First, high-quality datasets with large volumes are essential for providing the generalizability of the performance of AI algorithms. In the category of nonneoplastic pathology, datasets representing the more prevalent findings, such as those described above, can be obtained. However, rare findings such as worms, malignant melanoma, and intussusception, to name a few, can be more difficult to document with enough material for the development, training, and validation of new AI models.

## DOMAIN. Management of Pathology

### AI Task 4.1—AI-assisted Risk Stratification for Downstream Management after CCE

The framework for AI-assisted risk stratification for downstream management after CCE is summarized in Table 4.1

**Table 4.1:** AI-assisted risk stratification for downstream management after CCE.

Technique	CCE.
Domain	Management of pathology.
AI task	Risk stratification for downstream management after CCE.
Description	AI-supported assessment of patient demographics, clinical risk factors, and CCE findings to allocate patients to colonoscopy, sigmoidoscopy, biopsy, endoscopic resection, surgery, surveillance, or follow-up CCE.
Performance measure	Assessment of the detected lesions integrated with patients’ characteristics and the correct selection of follow-up strategy based on the current guidelines.
Rationale	AI may support consistent allocation of patients after CCE by integrating patient risk factors with lesion size, number, morphology, localization, and procedural adequacy. The aim is to guide appropriate downstream action while avoiding missed significant pathology and unnecessary procedures; this requires prospective validation in the intended clinical pathway.
Reference Standard	Experienced clinician/endoscopist panel applying current guidelines to CCE findings and downstream colonoscopy/histopathology where available. PIVI/SODA thresholds are used only as indirect safety benchmarks from colonoscopy optical-diagnosis research, not as directly transferable CCE criteria.
Expected value	AI-assisted allocation of patients to subsequent procedures or surveillance/follow-up should be comparable to expert clinical decision-making. Where leave-in-situ or follow-up strategies are considered, safety should be benchmarked against high NPV/sensitivity targets and prospectively validated.

**STATEMENT**

**For acceptance of AI-assisted downstream management after CCE findings to allocate patients for subsequent procedures, including colonoscopy, sigmoidoscopy, biopsy, EMR/ESD, or follow-up, AI-assisted assessment should be comparable to that of experienced colonoscopists NPV and sensitivity at or above the established PIVI/SODA standard targets.**

Agreement: 96.8%, at the first voting round.

## Discussion

After CCE, the central clinical question is not optical diagnosis in the colonoscopy sense, but allocation of patients to the appropriate next step. This requires integration of lesion size, number, morphology, location, bowel preparation, patient risk factors, symptoms, and pathway context. Because CCE cannot provide histology or immediate therapy, AI-supported management should primarily assist triage to colonoscopy, sigmoidoscopy, biopsy, endoscopic resection, surgery, surveillance, or follow-up CCE.

Such tools could reduce variation in decision-making and support more consistent application of guideline-based pathways, but they should not be treated as substitutes for specialist clinical judgment. Their clinical value will depend on transparent outputs, local validation, and demonstration that they improve allocation without increasing missed significant pathology or unnecessary downstream procedures.

Reference standards for this task should therefore include expert clinician adjudication and, where available, downstream colonoscopy and histopathological confirmation rather than direct transfer of optical-diagnosis benchmarks developed for conventional colonoscopy.

PIVI and SODA thresholds were developed for colonoscopy-based optical diagnosis strategies, including leave-in-situ and resect-and-discard approaches. They are therefore not directly transferable to CCE, which cannot biopsy, resect, provide histology, or perform optical diagnosis in the colonoscopy sense. In this position statement, PIVI and SODA are used only as indirect safety benchmarks for thinking about downstream management after CCE, particularly whether patients should proceed to colonoscopy, sigmoidoscopy, biopsy, endoscopic resection, surgery, surveillance, or follow-up CCE. Any AI-supported CCE management strategy that proposes deferring colonoscopy would require high negative predictive value, high sensitivity for clinically significant pathology, transparent decision logic, and prospective validation in the intended referral population.

Future advancements should focus on developing multiclass detection algorithms capable of not only identifying various abnormalities but also distinguishing those with significant clinical implications, resulting in recommendations according to guidelines in force.

## DOMAIN. Management of Pathology

### AI Task 4.2—AI-assisted Prediction of Submucosal Invasion as a Long-term Research Objective

The proposed framework for AI-assisted prediction of submucosal invasion is summarized in Table 4.2 **.**

**Table 4.2:** AI-assisted prediction of submucosal invasion as a long-term research objective.

Technique	CCE.
Domain	Management of pathology.
AI task	Prediction of submucosal invasion (long-term research objective).
Description	Exploratory AI-assisted prediction of submucosal invasion in colorectal lesions using CCE image-analysis algorithms. The goal is to support risk stratification and prioritization for colonoscopy, histology, endoscopic therapy, surgery, or multidisciplinary review, not to bypass established diagnostic steps.
Performance measure	Diagnostic accuracy in predicting submucosal invasion; concordance with histopathological outcomes; impact on treatment decision concordance.
Rationale	Submucosal invasion usually requires histological confirmation and may require surgery rather than endoscopic treatment. AI based on capsule images may eventually support early noninvasive risk stratification, but this remains highly speculative and requires well-characterized datasets with colonoscopic and histopathological correlation.
Reference Standard	Histopathological confirmation after colonoscopy, endoscopic resection, or surgery, with expert lesion characterization where appropriate.
Expected value	Exploratory aspirational value: ≥90% sensitivity and ≥90% specificity in well-characterized datasets. This threshold should not guide management without prospective validation and histopathological correlation.

**STATEMENT**

**For acceptance of AI in predicting submucosal invasion, the system must demonstrate diagnostic accuracy comparable to histopathological confirmation, with ≥90% sensitivity and specificity in well-characterized datasets.**

Agreement: 96.8%, at the first voting round.

## Discussion

The ability to accurately predict submucosal invasion in colorectal lesions is a critical determinant of appropriate therapeutic strategy. Lesions confined to the mucosa may be amenable to endoscopic resection, while those with submucosal involvement often necessitate surgical intervention due to increased risk of lymphovascular spread. Traditionally, this assessment has required either high-definition endoscopic evaluation with advanced imaging modalities (e.g., dye or virtual chromoendoscopy, magnification) or definitive histopathological analysis following tissue acquisition. However, these approaches are not feasible within the constraints of CCE, which remains a noninvasive, passive modality without tissue acquisition capability. 118 These proposed thresholds should therefore be interpreted alongside the broader literature on advanced imaging, AI implementation, capsule-endoscopy polyp recognition, optical diagnosis, CCE screening performance, and computer-aided polyp detection/classification. 118 119 120 121 122 123 124 125 126 127 128 129 130

The integration of AI into this part of the CCE pathway should be considered a long-term research opportunity rather than an implementation-ready clinical application. Unlike colonoscopy, CCE cannot insufflate, wash, biopsy, magnify, apply chromoendoscopy, or perform therapy, and capsule images may be affected by motion, partial views, residual stool, and variable illumination. These limitations make prediction of submucosal invasion from CCE substantially more speculative than lesion characterization during high-definition colonoscopy.

However, the challenge lies in validating these AI models against the current gold standard, histopathological assessment. High sensitivity is needed to minimize false negatives that could delay necessary surgery, while high specificity is required to avoid unnecessary escalation of care for benign lesions. The ≥90% sensitivity and specificity values proposed here should therefore be regarded as aspirational values in well-characterized datasets, not as thresholds for clinical management without prospective validation.

If eventually validated, AI-supported prediction of high-risk invasive features may help prioritize urgent therapeutic colonoscopy or surgical review, rather than bypassing the established steps of colonoscopic assessment, histological confirmation, and multidisciplinary decision-making. At present, this task should be regarded as hypothesis-generating and suitable for research datasets with robust colonoscopic and histopathological correlation.

Future research should focus on the development of large, annotated training datasets with histologically confirmed endpoints, stratified by lesion morphology, location, and clinical context.

## DOMAIN. Complications

### AI Task 5.1—AI-assisted capsule retention detection

The expected role and performance requirements for AI-assisted capsule retention detection are summarized in Table 5.1 **.**

**Table 5.1:** AI-assisted capsule retention detection.

Technique	CCE.
Domain	Complications.
AI task	CCE Retention.
Description	AI-assisted detection of capsule retention using pattern recognition algorithms on CCE image sequences to improve diagnostic accuracy, reduce the need for radiological investigations, and enable immediate follow-up planning.
Performance measure	Diagnostic accuracy for retention detection, concordance with radiological imaging, and reduction in unnecessary imaging procedures.
Rationale	The development of CNN for strictures identification presents complexity, given the impracticality of a frame-by-frame assessment. AI-assisted detection of CCE retention may help in the accurate diagnosis of this complication, avoiding the need for radiological techniques (reducing costs) and allowing immediate follow-up recommendations.
Reference Standard	Radiological imaging (e.g., CT scan) confirming capsule retention.
Expected value	Minimum expected value: AI-assisted reader assessment is superior to the reference standard. Aspirational value: ≥95% sensitivity and ≥95% specificity in identifying retention events.

**STATEMENT**

**For acceptance of AI in detecting capsule retention, the system must demonstrate diagnostic accuracy higher than radiological confirmation, with at least 95% sensitivity and specificity in well-characterized datasets**

Agreement: 91.7%, at the first voting round.

## Discussion

Capsule retention 131 is a rare but clinically significant complication of CCE, often associated with strictures or obstructive pathology, particularly in patients with known or suspected Crohn’s disease. 132 133 134 135 Early and accurate detection of retention is essential to prevent adverse events and to guide further diagnostic or therapeutic action. 136 Moreover, when retention is suspected, additional radiological studies (e.g., CT) are often ordered to confirm the location of the capsule, adding cost and complexity, 136 Table 5.1.1 . AI, particularly deep learning models like CNNs, offers potential for real-time or postprocessing detection of capsule retention by identifying features of luminal narrowing, lack of capsule progression, or recurring visual patterns over prolonged periods. 110 The only published study addressing AI-based retention detection 137 demonstrated that a neural network trained on a large image bank of Crohn’s disease cases could distinguish strictures from normal mucosa with an AUC of 0.989, achieving 92% sensitivity and 89% specificity.

**Table 5.1.1:** Sensitivity and specificity of capsule retention by radiologic image modality.

Imaging Modality	Sensitivity (Detection)	Specificity (Detection)
Plain Abdominal Radiograph	60–85%	80–95%.
CT scan (noncontrast or contrast-enhanced)	85–95%	90–98%.

This suggests that with appropriate training datasets, AI could be integrated into CCE review software to automatically flag suspected areas of concern, streamlining clinician review and reducing time to diagnosis. Such systems should be validated not only in Crohn’s cohorts but also in routine clinical screening and polyp detection populations. 138

Output from AI systems should include flagged frames, visual cues (e.g., bounding boxes), and probability scores. These features would enable clinicians to verify AI suggestions and reduce dependence on radiology in straightforward cases. Moreover, by identifying repeated image patterns and correlating transit time anomalies, AI can provide both confirmation of stasis and clues to its anatomical location.

Further studies are needed to assess the generalizability of retention-detection algorithms across capsule platforms and in mixed patient populations. Ethical implications, such as false reassurance from missed retentions or false positives leading to unnecessary interventions, must also be considered. Overall, the integration of AI in detecting CCE retention presents an important step toward safer, faster, and more cost-effective postprocedural care, particularly in high-risk groups or settings with limited access to imaging.

## DOMAIN. Complications

### AI Task 5.2—AI-assisted detection of mucosal compromise potentially associated with impending colonic perforation

The proposed framework for AI-assisted detection of colonic perforation is summarized in Table 5.2

**Table 5.2:** AI-assisted detection of mucosal compromise potentially associated with impending colonic perforation during CCE.

Technique	CCE.
Domain	Complications.
AI task	Early detection of mucosal compromise is potentially associated with colonic perforation.
Description	AI-assisted analysis of CCE frames to identify mucosal features (e.g., wall discontinuity, major localized defects, air-fluid interface anomalies) suggestive of early perforation.
Performance measure	Frame-level detection accuracy; correlation with subsequent (radiology) imaging or clinical findings.
Rationale	Full-thickness colonic perforation is a typically acute event, rarely (if ever) associated with CCE. However, AI may support early identification of precursor signs (such as focal mucosal breakdown or abnormal gas distribution patterns), particularly in surveillance of high-risk patients (e.g., post-GI surgery, strictures).
Reference Standard	Confirmed by cross-sectional imaging (CT scan), and/or subsequent surgical findings and/or clinical course.
Expected value	High NPV; clear flagging of frames of concern to support timely clinical reassessment and escalation when needed.

**STATEMENT**

**AI systems designed to detect mucosal compromise potentially associated with colonic perforation on CCE must demonstrate clinically meaningful accuracy, provide explainable output, and be validated against radiological or surgical confirmation** .

Agreement: 83.3%, at the first voting round.

## Discussion

The reliable identification of complications during CCE remains one of the most challenging and underexplored domains for AI. 110 Among these, colonic perforation, although very rare in the context of CE, is a critical event requiring rapid recognition and intervention. 133 134 Traditionally, this diagnosis relies on radiologic imaging (e.g., upright abdominal radiography or CT), which are both fast and definitive. Consequently, relying on CCE to detect an overt perforation may be clinically impractical and diagnostically unnecessary.

However, AI may still play a valuable role, not in diagnosing full-thickness perforation per se, but in early detection of subtle mucosal disruptions or pre-perforation indicators. 110 This may include detection of localized mucosal defects, aberrant intraluminal air patterns, focal areas of major wall thinning, or discontinuity. These features, while easily missed during manual review, may be detectable using DL algorithms trained to flag atypical or nonphysiological mucosal appearances. The potential here lies in early detection during high-risk clinical contexts, such as post-polypectomy surveillance, IBD, or postoperative capsules. In such cases, early AI-driven alerts could prompt timely confirmatory imaging or urgent clinical assessment, thus enhancing the success of conservative treatments and reducing the morbidity associated with complications.

A systematic literature review conducted using PubMed and Embase identified 333 articles, but none directly addressed AI-based detection of colonic perforation via CCE. 110 This notable absence highlights a significant evidence gap, suggesting that current AI applications in CCE are almost exclusively focused on the detection of polyps, bleeding, or cleansing quality, not safety or complications. From a development perspective, datasets annotated with perforation-related findings are exceedingly rare, as most clinical perforations are not captured on CE due to rapid progression or patient instability. Consequently, meaningful algorithm training requires synthetic data generation, multicenter aggregation of rare cases, or prospective surveillance of high-risk cohorts.

Until such evidence becomes available, the use of AI in this context should be considered investigational. Any real-time AI alerts related to suspected mucosal compromise should be:

- Explainable, with highlighted frames and classification rationale
- Reviewed by a qualified clinician
- Integrated with safety protocols, prompting urgent clinical and radiographic follow-up when needed

This underscores a broader principle: while AI can support efficiency and accuracy in routine diagnostics, its deployment in safety-critical tasks demands extreme caution, transparency, and human oversight.

## DOMAIN. Patient Experience

### AI Task 6—AI-assisted Capture, Recording, and Analysis of Patients’ Experience before, during, and Post CCE

The proposed framework for AI-assisted capture of patient experience is summarized in Table 6 .

**Table 6:** AI-assisted patient experience during the CCE journey.

Technique	CCE.
Domain	Patient experience.
AI task	Capture, record, extract, and analyze the patient’s CCE journey.
Description	Implement an AI-powered, automated pre-capsule endoscopy survey to assess patient concerns, perceived knowledge gaps, bowel prep experience, and anxiety levels related to the procedure. During the procedure, deploy a voice-to-text AI system to capture patients' verbal feedback, automatically transcribing and analyzing their experiences in real time. Postprocedure, an AI-driven chatbot survey systematically collects data on patient discomfort, the delayed side effects, and overall satisfaction, enabling clinicians to identify trends, optimize care pathways, and enhance patient-centered endoscopy experiences.
Performance measure	Efficient and rapid patient experience capture: The AI system must collect patient feedback seamlessly in a timely and efficient manner to support real-time analysis and decision-making. Targeted reevaluation of specific journey phases: The system should allow clinicians to reassess specific aspects of the patient journey, such as patient-reported bowel cleansing duration, enabling a focused review of key procedural steps. Standardized and reproducible output: AI-generated patient experience reports should provide consistent, structured, and comparable insights to facilitate benchmarking and evaluation. Accessible and inclusive design: The AI-driven information capture system should be patient-friendly, ensuring ease of use for individuals with low digital literacy or limited access to advanced technology. AI-enhanced patient communication: AI-generated outputs, such as personalized instructional guides or educational leaflets, must be clear, easy to understand, and tailored to diverse patient populations, enhancing engagement and compliance.
Rationale	The patient experience journey in CCE remains largely unexamined in the literature. There is a critical need to systematically capture and analyze patient feedback across the entire care pathway, including: Decision-making regarding lower GI investigations. Consent process and information provision. Preprocedural preparation and expectations. Intra-procedural experience. Post-procedural care, follow-up, and patient concerns. AI-driven data capture must be rapid, efficient, and seamlessly integrated into the patient journey. The AI-generated outputs should be standardized, reproducible, and adaptable for clinicians, allowing for benchmarking and comparison across different healthcare settings. Additionally, the AI system must be capable of producing accurate, patient-friendly reports and tailored informational materials that are clear, accessible, and easily understood by diverse patient populations, ultimately enhancing engagement, compliance, and overall patient experience in CCE.
Reference Standard	The EQ-5D instrument, which was used in colonoscopy studies to measure health-related quality of life (HRQoL), could be adapted for CCE to provide standardized data. 141 The combination of standardized HRQoL measures and meta-analytic techniques can provide a deeper understanding of the patient experience in CCE and inform strategies to improve clinical practice.
Expected value	Minimum expected values: reliability ≥90% and transparency ≥95%. Aspirational values: reliability ≥95% and transparency 100%, data generation without compromising patients’ privacy.

**STATEMENT**

**AI-assisted evaluation of patient experience in CCE should be comparable to standard (non-AI) CCE and/or conventional colonoscopy**

Agreement: 86.4%, at the second voting round.

## Discussion

In healthcare, patient experience should always be a priority, yet it often receives insufficient attention due to the overwhelming demands of managing waiting lists and addressing clinical concerns. Capturing and analyzing patient experience in a structured and meaningful way remains a challenge, as traditional methods are time-consuming and often deprioritized in routine practice. Existing literature on patient experience in CCE primarily focuses on comparisons with colonoscopy. 139 A systematic review has examined CCE tolerability, patient-reported outcomes, and preferences, 140 but the scope has been largely limited to intraprocedural experiences. Crucial preprocedural factors, such as shared decision-making between CCE and colonoscopy, dietary preparation (low-residue diet), and concerns regarding potential complications, are poorly captured in research and in daily clinical practice. With advancements in AI, particularly large language models, it is now possible to systematically capture, analyze, and present patient feedback in real time. AI-powered analysis of patient surveys can provide structured insights, identifying key areas for improvement and enhancing patient-centered care. Moreover, AI-driven patient experience metrics could serve as a key performance indicator (for instance, achieving patient satisfaction rates above 80%). The components of patient satisfaction would require further research, with appropriate weighting assigned to different factors. Since AI enables instant data collection, storage, and analysis, it could establish standardized benchmarks for patient-centered services, ultimately driving continuous improvement in endoscopy care.

## DOMAIN. Postprocedure

### AI Task 7.1—AI-supported urgency categorization of the CCE report

The framework for AI-supported categorization of CCE reports according to clinical urgency is summarized in Table 7.1 .

**Table 7.1:** AI-supported urgency categorization of CCE reports.

Technique	CCE.
Domain	Postprocedure.
AI task	To correctly categorize CCE reports based on clinical urgency.
Description	AI-supported categorization of CCE report based on clinical urgency.
Performance measure	Rate of correct categorization of CCE-reports.
Rationale	To develop a high-performing model that can evaluate the patient report, demographic, comorbidity, biomarkers (FIT), and blood parameters, and thereby categorize the patients by need for urgent clinical assessment. Guiding clinicians’ decisions for the appropriate management of the individual patient. **Urgent (Red)** —would be a cancer found by CCE, or even high biomarker patients with incomplete CCE, because of their still high need for investigation, would prompt the clinician to organize an urgent alternative colonic investigation. **Nonurgent (Amber)** —findings suitable for the locally defined nonurgent pathway. **Routine (Green)** —complete, adequately prepared CCE with no clinically significant findings and no indication for further investigation.
Reference Standard	Clinician-validated categorization based on full review of the CCE report, clinical data (demographics, comorbidities), laboratory biomarkers (including FIT), and the subsequent clinical action taken.
Expected value	Correct categorization of reports in ≥90% with an aspirational value of ≥95%.

**STATEMENT**

**For acceptance of AI-supported categorization of CCE reports based on clinical urgency, the categorization should be comparable to the urgency evaluations made based on findings of colonoscopy.**

Agreement: 91.7%, at the first voting round.

## Discussion

Establishing a robust and clinically valid reference standard is critical for evaluating the performance of AI systems tasked with categorizing CCE reports based on urgency. 9 In this context, the reference standard must reflect real-world clinical decision-making, integrating objective findings from the CCE report and contextual patient data. 76

The most appropriate benchmark is the final clinical categorization assigned by expert reviewers or a multidisciplinary team after full review of:

- CCE findings (e.g., polyp characteristics, completeness of study)
- Patient demographics
- Comorbidities (e.g., IBD, prior CRC, iron deficiency anemia)
- Laboratory results (e.g., FIT values, hemoglobin, ferritin)

This clinician-driven decision represents the current gold standard against which AI performance should be measured. Importantly, it reflects both diagnostic insight and patient management needs, not simply the image-based findings. For example, a patient with an incomplete CCE but a FIT >150 μg/g would likely be triaged as “urgent,” regardless of visualized pathology. Moreover, because clinical decisions often vary depending on healthcare setting, available resources, and national guidelines, the reference standard should ideally be calibrated against actual clinical actions taken, such as whether a patient was referred for colonoscopy within two weeks, placed on an elective waitlist, or discharged. This outcome-based validation grounds the AI model in practical utility rather than purely theoretical accuracy.

To ensure consistency, the reference standard should be defined in advance through a standardized protocol, ideally involving:

- Two or more independent expert reviewers
- A consensus mechanism for discordant cases
- Retrospective matching of AI-generated urgency labels to clinician-validated labels

Evaluating agreement using metrics such as accuracy, Cohen’s kappa, and false negative rate for urgent cases is essential. Notably, failure to identify “Red” category cases represents a clinically unacceptable error.

## DOMAIN. Postprocedure

### AI Task 7.2—AI-generated CCE Report

The expected standards for AI-generated CCE reporting are summarized in Table 7.2 **.**

**Table 7.2:** AI-generated CCE report.

Technique	CCE.
Domain	Postprocedure.
AI task	To correctly generate CCE reports.
Description	AI-supported generation of a CCE report.
Performance measure	Accuracy and completeness of AI-generated CCE reports.
Rationale	To develop a high-performing model that can generate patient and clinician reports of the CCE examination that will include: Relevant symptomatic history and patient demographics, frailty, relevant biomarkers, etc. Relevant information about complete transit, segments successfully examined, and bowel preparation. Relevant pictures from the examination, and choosing the best picture taken of the pathology or segments to illustrate the report.
Reference Standard	Clinician consensus.
Expected value	Correct generation of reports in ≥95% of cases, with an aspirational value of 100%.

**STATEMENT**

**For acceptance of AI-generated CCE reports, their accuracy, completeness, and clinical usability should be comparable with clinician-generated reports.**

Agreement: 95.8%, at the first voting round.

## Discussion

Reporting after procedures is among the most labor-intensive and variable aspects of the CCE workflow. This process often consumes 45–60 minutes per case, depending on capsule transit time, complexity of findings, and reader experience. 111

Currently, the reporting process relies entirely on human judgment and is subject to considerable interobserver variability, particularly in determining lesion location, assessing bowel preparation, and characterizing lesion morphology. Moreover, the impact of reader fatigue has been repeatedly demonstrated in CE, where prolonged reading sessions result in reduced sensitivity, particularly for small or flat lesions. 76

AI is well-positioned to improve this aspect of clinical practice. By automating the assembly of findings into structured postprocedure reports, AI can:

- **Generate draft postprocedural reports** by pre-filling key elements (e.g., lesion type, location, size)
- **Standardize report content** across centers and readers
- **Improve consistency** in prep grading and lesion descriptions
- **Enable triage** by categorizing reports based on urgency or abnormality

Such automation does not replace clinical judgment; rather, it enhances it. The clinician would review and edit the draft, validating the findings, adding context, and signing off.

So far, no studies have validated AI-generated reports in routine clinical practice. However, foundational components already exist: AI models have been developed for polyp detection, segment identification, cleansing assessment, and size estimation. Integrating these into a unified reporting pipeline is the logical next step. Lessons from colonoscopy AI (e.g., GI Genius) and SBCE systems show that automated summaries can be accurate, efficient, and well-accepted.

In the broader system context, AI-generated reports could also:

- Feed structured data into hospital electronic medical records
- Support national screening audits
- Provide patients with simplified summaries for informed follow-up

## DOMAIN. Ethics

### AI Task 8—AI-related Ethics Issues in CCE

The principal AI-related ethical issues relevant to CCE are summarized in Table 8 **.**

**Table 8:** AI-related ethics issues.

Technique	CCE.
Domain	AI-related ethics issues in CCE.
AI task	To be ethically acceptable.
Description	Ethical considerations are to be integrated into implementation at all levels. Issues that need to be considered and addressed include potential short- and long-term benefits and harms, secondary findings, bias, explainability, and the impact of AI on the clinician’s role—including the risks of overreliance and deskilling—and on the doctor–patient relationship.
Performance measure	Awareness of ethical issues and guidelines needs to be implemented as part of planning, routine, and preparedness for arising issues to maintain patient trust in health care systems and staff.
Rationale	There are numerous guidelines, including ethical ones, by expert groups such as the European Union (EU) 142 and the World Health Organization. 143 However, there is very little literature on the issue of ethics and CCE. Mascarenhas M et al. (2023) 144 is an exception. Many of the issues highlighted in these guidelines emphasize the more technical ethical aspects of AI. Additionally, the ethics surrounding endoscopy remain underdeveloped, and the field of AI ethics is still emerging.
Reference Standard	Best medical practice in terms of ethics (biomedical principles). The relevant national and professional medical guidelines (or the closest relevant one, e.g., AI in radiology). The EU guidelines for Trustworthy AI (for AI ethics). The relevant national and/or regional legal AI requirements (e.g., General Data Protection Regulation -GDPR, EU law on medical devices, EU AI Act).
Expected value	Ethical awareness across implementation (including health staff and doctors): this includes moral literacy and awareness of the ethical developments in the field. Broader contextual issues may need to be considered for overall ethical assessment.

**STATEMENT**

**The ethical standards governing AI-assisted CCE procedures should meet or exceed current standards of medical practice.**

Agreement: 95.8%, at the first voting round.

## Discussion

AI-assisted CCE, as a viable alternative to conventional colonoscopy, has the potential to substantially reduce screening wait times and enable earlier detection of CRC. It may also be more acceptable to many patients due to its less invasive nature. AI in CCE must adhere to principles of beneficence, nonmaleficence, justice, and autonomy, as outlined in global frameworks. The ethical justification for implementing AI-assisted CCE will largely depend on its overall performance and its broader impact on national and local healthcare systems, with implications extending to society at large. Key considerations include patient trust, quality of care, clinical effectiveness, patient experience, and the confidence and preferences of healthcare professionals. Additionally, economic factors, such as cost-effectiveness and the sustainability of healthcare expenditures, must be considered, particularly given the global rise in healthcare costs.

To be ethical, solutions must align with legal requirements and be sound in both biomedical and technical aspects. Additionally, they should be technically robust. Regulation (EU) 2024/1689 (the EU AI Act) addresses some of these challenges. 142 It is also necessary that solutions are robust and future-proof, so they will not have to be replaced prematurely. 143 The biomedical requirements that solutions need to align with are Autonomy, Beneficence, Non-Maleficence, and Justice, all requirements that medical doctors have been taught for generations and see as the hallmark of good medical practice. 142 143

AI ethical requirements include Human Autonomy, No Harm, Fairness, and Explicability, which should also be a way of avoiding bias. These are, to some degree, overlapping with the biomedical principles. Ethical implementation of solutions needs to include ethical consideration at all points of implementation, from overall planning and purchasing through the entire pathway, and to follow-up as part of an ethical culture. 144 145 This will include training and communication between both health staff and technical specialists.

It is also necessary in the implementation process to consider epistemic obstacles arising from the present black-box challenges: for computer engineers to “health-check” technology, for doctors to assess outcomes, and for patients to be able to give/withhold consent. 144 145 We suggest that clinicians and AI specialists work together to establish a common understanding of ethical expectations from existing guidelines and shared terminology. Without such an understanding, they are bound to talk past each other, which will lessen trust in AI and threaten patient safety and health systems’ robustness. 142 143

The ethics of AI-assisted CCE must meet or exceed current medical ethical standards (beneficence, non-maleficence, autonomy, justice) and comply with the EU AI Act, GDPR, and WHO guidance. Key issues requiring proactive governance include: liability for AI misses, risk of deskilling, algorithmic bias in underrepresented populations, explainability for clinicians and patients, and equitable access/reimbursement. Institutions implementing AI-CCE should establish local oversight committees.

## Conclusions

This position statement proposes consensus-based expected-value benchmarks and normative considerations for the use of AI in CCE. The task force was created within the AICE consortium project work and drew on members of the iCARE group for development, review, consensus voting, and approval.

The integration of CCE into CRC prevention strategies is justified by the need to complement, rather than replace, existing diagnostic approaches, which are predominantly reliant on colonoscopy. CCE offers a minimally invasive modality for colonic evaluation and is particularly relevant in clinical settings where access to colonoscopy is limited or delayed, where colonoscopy is contraindicated, or where it is declined by patients. Although CCE has demonstrated the capability to detect clinically relevant colonic pathology, its broader adoption in routine clinical practice has been constrained by several limitations, including time-consuming manual video review, substantial interobserver variability, and challenges related to procedural completeness, anatomical localization, and reliable attribution of findings.

The incorporation of AI is expected to fundamentally change this paradigm by enabling standardized, reproducible, and scalable assessment of key procedural parameters. This transformation has the potential to convert CCE from a reader-intensive diagnostic test into a structured, auditable clinical decision-support tool. AI-supported CCE may facilitate risk stratification and diagnostic resolution, for example, by triaging patients, identifying individuals who require therapeutic colonoscopy, and safely excluding clinically significant pathology in selected cases, while preserving colonoscopy capacity for patients most likely to benefit from invasive endoscopic intervention.

With respect to lesion detection and characterization, we propose ambitious but explicitly provisional targets. Many of these remain aspirational and should be tested in large, prospective, externally validated, full-video CCE studies before they are used as clinical adoption requirements. In selected domains, such as completeness of mucosal visualization, rapid video review, lesion sizing and localization, and reporting, AI may eventually improve standardization and reproducibility, but implementation must remain tied to clinical safety, interpretability, and clearly defined reference standards.

Importantly, AI-supported CCE is not intended to eliminate the need for clinical oversight or professional accountability. Its implementation must remain fully aligned with established principles of responsible, human-centered AI in clinical medicine, as well as with evolving regulatory and ethical frameworks.

## Current Evidence Gaps and Limitations

Current evidence remains insufficient for the routine clinical implementation of most AI-assisted CCE tasks, and several reference standards are themselves imperfect. The thresholds proposed here are expected-value benchmarks and research priorities, not mandatory clinical requirements. Most are extrapolated from colonoscopy performance measures, CE literature, small pilot studies, or expert consensus. Prospective clinical validation, external validation across platforms and populations, and future evidence synthesis will be required before these thresholds can be converted into formal guideline recommendations.

**Abbreviations**
AI: Artificial Intelligence
AICE: Artificial Intelligence in Capsule Endoscopy
AUC/AUROC: area under the curve/area under the receiver operating characteristic curve
CE: Capsule endoscopy
CCE: Colon Capsule Endoscopy
CESCAIL: Capsule Endoscopy delivery at sCale through enhanced AI analysis
CNN: Convolutional Neural Network
CRC: Colorectal Cancer
DL: deep learning
EMR: Endoscopic Mucosal Resection
ESD: Endoscopic Submucosal Dissection
ESGE: European Society of Gastrointestinal Endoscopy
EQ-5D: EuroQol-5D
FIT: Fecal Immunochemical Test
FPR: False Positive Rates
GDPR: General Data Protection Regulation
GI: Gastrointestinal
HRQoL: health-related quality of life
IBD: Inflammatory Bowel Diseases
iCARE group: international Capsule Endoscopy Research group
LSTM: Long short-term memory
ML: machine learning
NPV: Negative Predictive Value
PCE: pan-enteric CE
PIVI: Preservation and Incorporation of Valuable Endoscopic Innovations
PPV: Positive Predictive Value
SBCE: Small Bowel Capsule Endoscopy
SSLs: Sessile Serrated Lesions
ScotCap: The Scottish Capsule Program
SLAM: Simultaneous Localization and Mapping
SODA: Simple Optical Diagnosis Accuracy
